# Response surface optimization of pudding formulation containing fish gelatin and clove (*Syzygium aromaticum*) and cinnamon (*Cinnamomum verum*) powder: Effect on color, physicochemical, and sensory attributes of the final pudding product

**DOI:** 10.1002/fsn3.2761

**Published:** 2022-02-26

**Authors:** Nasrin Choobkar, Amir Daraei Garmakhany, Abdolraza R. Aghajani, Maryam Ataee

**Affiliations:** ^1^ Department of Fisheries Faculty of Agriculture Kermanshah Branch Islamic Azad University Kermanshah Iran; ^2^ Department of Food Science and Technology Toyserkan Faculty of Engineering and Natural Resources Bu‐Ali Sina University Hamedan Iran; ^3^ Department of Food Science and Technology Faculty of Industrial and Mechanical Engineering Qazvin Branch Islamic Azad University Qazvin Iran; ^4^ Department of Food Science and Technology, Science and Research Tehran branch Islamic Azad University Tehran Iran

**Keywords:** cinnamon, clove, fish gelatin, pudding, response surface methodology

## Abstract

In this study, the response surface methodology (RSM) was used to optimize the pudding formulation ingredients including the fish/bovine gelatin ratio and cinnamon and clove powder and determine the color and physicochemical and sensory attributes’ change in final pudding product. Experiments were carried out based on a central composite design (CCD). The results showed that by increasing the ratio of fish gelatin to bovine gelatin (FG/BG) up to 3%, the moisture content increased slightly and then decreased significantly. Increasing the cinnamon powder to 0.5% reduced the moisture content. Increasing the FG/BG in the formulation of pudding samples reduced the protein content. The effects of cinnamon and clove powder on the protein content were increasing and decreasing, respectively. By increasing the FG/BG ratio, the samples syneresis showed a significant decrease, while the effects of cinnamon and clove powder on the syneresis were nonsignificant. As the level of cinnamon and clove powder increased, the L^*^ value decreased. Cinnamon and clove powder had a linear effect, and the interaction of gelatins and clove powder had a significant effect on changes in redness. The effects of cinnamon and clove powder on b^*^ value were significant. In terms of sensory evaluation, increasing the cinnamon powder concentration increased the appearance scores, while in the case of fish gelatin, this trend was downward. The linear effect of cinnamon powder on taste was significant, while other variables had no significant effect on the taste of the samples. The sample texture was significantly affected by fish gelatin and clove powder. Increasing FG/BG from 0% to 2.5% increased the texture score, but after this range, a decrease in the texture score was observed. The overall acceptance of samples was more affected by spice powder compared to gelatin. By increasing the cinnamon powder and FG/BG, the overall acceptance increased and decreased, respectively. In conclusion, the optimal FG/BG and cinnamon and clove powder were introduced 1.479%, 0.288%, and 0.619% respectively.

## Graphical abstract

In this study, the pudding formulation was optimized regarding the fish/cow gelatin ratio, cinnamon and clove powder, and physicochemical as well as sensory analysis. The optimal FG/BG and cinnamon and clove powder were introduced 1.479, 0.288, and 0.619%, respectively.

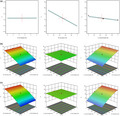

## 1 INTRODUCTION

Functional food contains some health‐promoting compounds beyond the traditional nutrients and plays an important role in enhancing human health (Sun et al., 2007). Dairy desserts are products of wide consumption. Their nutritional and organoleptic characteristics make them accepted by children and adults, but usually they have high energy density (Alimoradian et al., 2021; Tarrega & Costell, 2006). Pudding is one of the most important milk products, it is the semisolid milk protein, consisting of starch‐based pastes and dairy desserts, which is a milk‐based starch paste and has a typical semisolid food texture (Lim & Narsimhan, 2006). Pudding is dessert prepared by addition of suitable quantity of egg to whole milk or concentrated milk or condensed milk. If needed, any suitable sweetener and thickening materials could be added to the mixture (Sarker et al., 2016). Dairy desserts (milk puddings) are especially consumed by children and elderly people throughout the world. Pudding is characterized as a suspension of deformable particles (the swollen starch granules) dispersed in a continuous phase containing milk proteins and a gelling agent (Verbeken et al., 2006). Powdered and packaged forms of the pudding samples are available in the market (Toker et al., 2013). The formulation of the puddings is generally composed of vanilla, milk, sugar, starch, and gum (Ares et al., 2009) and the other ingredients such as cacao and fruit aromas, which are responsible for developing distinctive rich aroma of the puddings (Gurmeric et al., 2013). Due to these complex interactions among the different components of a pudding, it is likely that the substitution of milk with other dispersion media could cause drastic changes in the rheological behavior of the product. This aspect, however, has been little investigated in the literature (Lim & Narsimhan, 2006; Nunes et al., 2003). Besides ready‐to‐eat puddings, commercial powders are at consumers’ disposal for the production of home‐made desserts. Pudding powders are usually composed of starch, hydrocolloids, sugars, colorings, and aromas, and they are intended to be dissolved in milk (Vélez‐Ruiz et al., 2006).

Gelatin from bone and connective tissue of pigs and cattle is traditionally used in the food industry as a gelling agent (Kouhi et al., 2020). However, the consumption of gelatin from these mammalian species contradicts ethnocultural and religious norms of a number of religions, and is also associated with the risk of contracting prion (Ahmed et al., 2020). In this regard, it seems relevant to look for alternative sources of food gelatin (Huang et al., 2019). Such a source may be the connective tissue of fish (Lv et al., 2019), the industrial processing of which partially solves the problem of disposal and integrated use of waste from the fish processing industry (Uranga et al., 2020). There are several papers on the use of fish gelatin in food formulations (Giménez et al., 2013; Wangtueai et al., 2020) (Yasin et al., 2016). In our previous study, we successfully used fish gelatin instead of bovine gelatin in the formulation of replacement on pastille (Asgarzadeh et al., 2021).

Spices always play a prominent role in the kitchen as well as in certain medicinal activities like diuretic, eccoprotic, carminative aperients, expectorant, and many more. Spices have been used medicinally since early (Sachan et al., 2018). Utilization of spices in various forms like powder, extract, or essential oils has been well documented for inhibiting the growth of many spoilage bacteria and fungi in foods (Rajkumar & Berwal, 2003; Subbulakshmi & Naik, 2002). Clove (*Syzygium aromaticum*) is one of the most valuable spices that have been used traditionally as a food preservative and for many therapeutic purposes. Clove is native to Indonesia, but it has also been cultured in several parts of the world (Hussain et al., 2017); it is a vital source of phenolic compounds such as flavonoids, hydroxycinnamic acids, and hydroxybenzoic acids. Eugenol is the main bioactive constituent of clove. With regard to the phenolic acids, gallic acid is found in higher concentration (783.50 mg/100 g fresh weight) (Shan et al., 2005). Clove and essential oils are reported to have antibacterial, antimycotic, yeast inhibitor, and Brownian enzyme inhibitor activity (Aghajani & Daraei Garmakhany, 2021; Daraei Garmakhany et al., 2017; Ghahfarrokhi et al., 2013). Such activity may be attributed to its 2‐methoxy‐4‐(2‐propenyl) phenol content.

Cinnamon (*Cinnamomum verum*) is one of the most well‐known spices. It is used in the pharmaceutical and food industry as a powerful antiseptic and flavoring agent, respectively. It presents medicinal properties like digestive, stimulant, hypotensive, sedative, and vasodilator (Cemin, 2012). Cinnamon spice is one of the sources of effective antioxidants such as vanillic, caffeic, gallic, protocatechuic, p‐hydroxybenzoic, p‐coumaric, and p‐hydroxybenzaldehyde (Muchuweti, 2007) and enhances the efficacy of other important antioxidants. The antioxidant activity of cinnamon is attributed to an array of flavonoid compounds that it contains. The essential oils present in cinnamon, including cinnamaldehyde, eugenol, and linalool, have been investigated in reference to peroxynitrite‐induced nitration and lipid peroxidation (Iqbal et al., 2005).

Regarding dairy products, many studies have been published dealing with the effect of fat content on quality properties. However, only a few papers deal with the quality characterization of commercial dairy desserts or pudding under the effect of gelling agents such as protein and fish gelatin (Fan et al., 2019; Yusof et al., 2019). Optimization of ingredients to obtain the desired quality of the product or optimization of the process to achieve desired results is performed by various optimization methods such as mixture design, response surface method (RSM), genetic algorithm (GA), and artificial neural network (ANN) (Daraei Garmakhany & Aghajani, 2021; Daraei Garmakhany et al., 2021; Ghahfarrokhi et al., 2013; Kashiri et al., 2012; Toker et al., 2013). This optimization method includes more than one ingredient or independent variables that their effect on the dependent variables or response was examined (Dutcosky et al., 2006). The relationship between independent factors and responses is explained by predictive mathematical models obtained from optimization approach (Flores et al., 2010). The aim of this study was to develop a pudding formulation containing clove and cinnamon powder and bovine gelatin accompanied by fish gelatin.

## 2 MATERIAL AND METHODS

### 2.1 Materials

Ingredients used for pudding were purchase from local markets. All other chemicals used in this study were of analytical grade and purchased from chemical suppliers.

### 2.2 Preparation of the pudding samples

**FIGURE 1:**
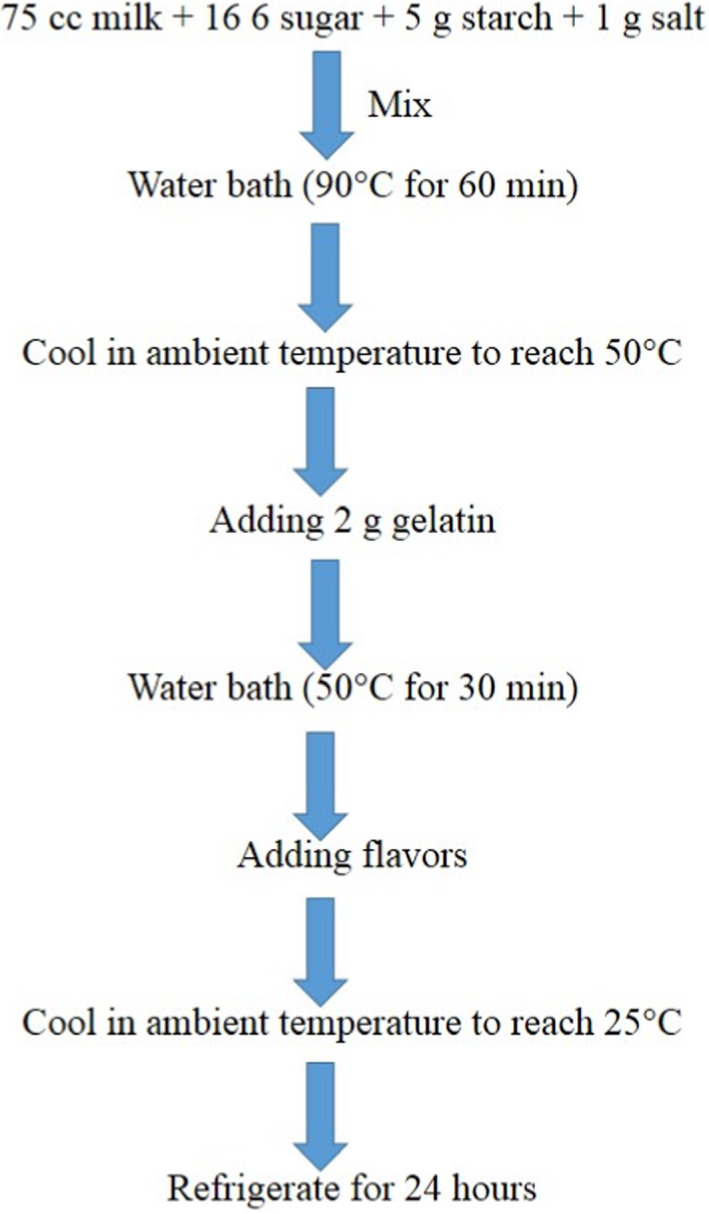
Flowchart of pudding production stages in this research

The formulation of the pudding includes 10 g sucrose and 9 g skimmed milk powder. The other ingredients include fish gelatin, bovine gelatin (0%, 2.5%, and 5%), and clove and cinnamon powder (0%, 0.5%, and 1%). The different treatment combinations used in the experimental design are shown in Table 1. Puddings were prepared by adding the solid mixture to 100 ml water slowly and mixing them with a magnetic stirrer (Yellow line, Germany). The dispersion was heated to 85°C for 20 min and stirred for 10 min at that temperature. The puddings were then cooled to room temperature (25°C) and then stored in a refrigerator (4–5°C) for 1 hr prior to the analysis. The experimental work was carried out in the Research Chemistry Laboratory, University of Tehran Science and Technology Park (Figure 1).

**TABLE 1:** Independent variable values of the process and their corresponding levels

Factor	Name	Units	Actual value		Coded value	
			Minimum	Maximum	Minimum	Maximum
A	Fish gelatin	%	0	5	−1	+1
B	Cinnamon powder	%	0	1	−1	+1
C	Clove powder	%	0	1	−1	+1

### 2.3 Experimental tests

#### 2.3.1 Moisture content

The moisture contents of different types of samples were determined by hot oven drying method according to AOAC (AOAC, 2003).

#### 2.3.2 Protein content

Protein percent of different pudding sample was determined by Kjeldahl procedure according to AOAC (2003).

#### 2.3.3 Syneresis percent

Ten grams of each sample were transferred to previously weighted centrifugation tubes and were held vertically at 4°C for 14 days. Syneresis was quantified as the loss of weight of the sample after the removal of the exuded water (Aichinger et al., 2003; Azari‐Anpar et al., 2017; Kokabi et al., 2021; Mehrinejad Choobari et al., 2021). The extent of syneresis was expressed as a percentage of exuded water as is referred in Equation 1:

(1) Syneresis(%)=exudedwater(g)/totalweightofthesample×100

#### 2.3.4 Color parameters (L^*^a^*^b^*^)

To measure the color parameters (L^*^, a^*^, and b^*^) of the samples, a wooden rectangular box with a floor area of 2400 cm^2^ with two 9‐watt white fluorescent lamps with an internal light intensity of 75 lux was used, and then the L^*^, a^*^, and b^*^ were measured. Finally, it was analyzed by Image J software (Daraei et al., 2021; Gohari Ardabli et al., 2021; Hashemi Shahraki et al., 2014).

#### 2.3.5 Viscosity measurement

Kay et al. (2017) method was used to determine the viscosity. For this purpose, 600 ml of the pudding was transferred to a beaker immediately after mixing. Viscosity was measured by a Brookfield viscometer with spindle 4 at speeds of 20 rpm (Kay et al., 2017).

#### 2.3.6 Sensory evaluation

The sensory evaluation of prepared pudding samples with different treatments was conducted by a trained panel of 10 judges. Each panelist was given a set of pudding separately in isolated chamber and provided with a glass of fresh water to rinse their mouth before tasting the next sample. Each sample was evaluated for various quality attributes, such as appearance, color, texture, mouthfeel, taste, and overall acceptance as per the prescribed proforma (Singh & David, 2017). Panelists were asked to rate the samples on a prescribed sensory evaluation proforma with earlier stated attributes. Samples were assessed organoleptically using a 5‐point hedonic scale, where 5 correspond to “like extremely” and 1 corresponds to “dislike extremely.”

### 2.4 Statistical analysis and experiment design

#### 2.4.1 The experiment design by RSM

In this study, the central composite design (CCD) was selected to optimize the process variables in two levels with 17 components, including three replicates at the central point for the estimation of the experimental error. The ranges and levels of independent variables are presented in Table 1. The statistical significance test was based on total error with 95% confidence level (*p* < .05). The independent variables of the process were included fish gelatin (A), cinnamon powder (B), and clove powder (C). According to Table 1, the ratio of fish gelatin to bovine gelatin varied between 0% and 5% of the sample weight, and the changes in cinnamon and clove powders were 0 to 1, which was calculated with alpha (α) equal to 1. The optimization of functional pudding production was done by using Design Expert software version 11.1.2.0 (Stat‐Ease Inc., Minneapolis, MN, USA) (Table 2). The multivariate model is an Equation (2). In the mentioned equation, Y is the predicted response, β0 is the constant coefficient, β_1_ and β_2_ are linear coefficients, β_11_ and β_22_ are square effects, and β_12_ and β_21_ are interaction effects.

(2) $$Y=\beta_{0}+\beta_{1}\left(A_{1}\right)+\beta_{2}\left(A_{2}\right)+\beta_{1}2\left(A_{1}A_{2}\right)+\beta_{1}1{\left(A_{1}\right)}^{2}+\beta_{2}2{\left(A_{2}\right)}^{2}$$

**TABLE 2:** The central composite design (CCD) and actual levels of independent variables for optimizing the functional pudding formulation

Run	Factor 1	Factor 2	Factor 3
	A: Fish gelatin (%)	B: Cinnamon powder (%)	C: Clove powder (%)
1	2.5	1	0.5
2	2.5	0.5	0.5
3	5	0.5	0.5
4	0	0	0
5	0	1	0
6	0	0	1
7	2.5	0	0.5
8	0	1	1
9	5	1	1
10	2.5	0.5	0
11	0	0.5	0.5
12	5	0	1
13	2.5	0.5	0.5
14	5	1	0
15	2.5	0.5	1
16	5	0	0
17	2.5	0.5	0.5

#### 2.4.2 Process optimization

The RSM evaluated the effects and interactions of the fish gelatin, clove and cinnamon powder to increase protein and sensory attributes, decrease syneresis while other parameters were in range. For validation of the correlation, the responses were also experimentally analyzed and compared with predicted values from the regression equation.

## 3 RESULTS AND DISCUSSION

### 3.1 Moisture content

Jelly is one of the high moisture foods (Hartel et al., 2018). The moisture content is important to determine the shelf life and purity of the protein (Baziwane & He, 2003). Low moisture content increases the product's stability, thus increasing the shelf life of the product (Chukwu & Abdullahi, 2015). Regarding the moisture content of the treatments, according to Table 3, the proposed model was not significant (*p* > .05), and the independent variables in linear, binomial, and quadratic had no effect on the model (Table 3).

**TABLE 3:** The analysis of variance of the predicted linear and quadratic polynomial models for predicting physicochemical properties of pudding formulation

Response	Source	Sum of squares	*df*	Mean square	*F*‐value	*p*‐Value
Moisture content	Model	2.08	6	0.3464	1.39	0.3084^ns^
	A‐Fish Gelatin	0.1490	1	0.1490	0.5963	0.4579^ns^
	B‐Cinnamon	0.0210	1	0.0210	0.0842	0.7776^ns^
	C‐Clove Powder	0.0602	1	0.0602	0.2409	0.6342^ns^
	AB	0.5948	1	0.5948	2.38	0.1539^ns^
	AC	0.8920	1	0.8920	3.57	0.0881^ns^
	BC	0.3617	1	0.3617	1.45	0.2566^ns^
	Residual	2.50	10	0.2498		
	Lack of Fit	0.9723	8	0.1215	0.1593	0.9771^ns^
	Pure Error	1.53	2	0.7630		
	Cor Total	4.58	16			
	*R* ^2^	0.4542				
	Adjusted *R* ^2^	0.1266				
Protein content.	Model	0.3799	3	0.1266	51.62	<0.0001*
	A‐Fish Gelatin	0.3765	1	0.3765	153.48	<0.0001*
	B‐Cinnamon	0.0034	1	0.0034	1.39	0.2592^ns^
	C‐Clove Powder	0.0000	1	0.0000	0.0000	1.0000^ns^
	Residual	0.0319	13	0.0025		
	Lack of Fit	0.0262	11	0.0024	0.8369	0.6608^ns^
	Pure Error	0.0057	2	0.0028		
	Cor Total	0.4118	16			
	*R* ^2^	0.9226				
	Adjusted *R* ^2^	0.9047				
Syneresis	Model	523.33	9	58.15	187.09	<0.0001**
	A‐Fish Gelatin	479.79	1	479.79	1543.74	<0.0001*
	B‐Cinnamon Powder	0.0004	1	0.0004	0.0014	0.9709^ns^
	C‐Clove Powder	0.0538	1	0.0538	0.1730	0.6899^ns^
	AB	0.1800	1	0.1800	0.5792	0.4715^ns^
	AC	0.3756	1	0.3756	1.21	0.3080^ns^
	BC	0.0556	1	0.0556	0.1788	0.6851^ns^
	A²	29.11	1	29.11	93.66	<0.0001*
	B²	0.0778	1	0.0778	0.2504	0.6322^ns^
	C²	0.0023	1	0.0023	0.0075	0.9332^ns^
	Residual	2.18	7	0.3108		
	Lack of Fit	1.18	5	0.2360	0.4741	0.7833^ns^
	Pure Error	0.9956	2	0.4978		
	Cor Total	525.50	16			
	*R* ^2^	0.9959				
	Adjusted *R* ^2^	0.9905				
Apparent viscosity	Model	7,323,000	9	813,700	1095.35	<0.0001*
	A‐Fish Gelatin	7,191,000	1	7,191,000	9679.98	<0.0001*
	B‐Cinnamon Powder	28,090	1	28,090	37.81	0.0005*
	C‐Clove Powder	34,810	1	34,810	46.86	0.0002*
	AB	200	1	200	0.2692	0.6198^ns^
	AC	1250	1	1250	1.68	0.2357^ns^
	BC	200	1	200	0.2692	0.6198^ns^
	A²	42,955.14	1	42,955.14	57.82	0.0001*
	B²	7.03	1	7.03	0.0095	0.9252^ns^
	C²	7.03	1	7.03	0.0095	0.9252^ns^
	Residual	5200.14	7	742.88		
	Lack of Fit	4733.47	5	946.69	4.06	0.2095^ns^
	Pure Error	466.67	2	233.33		
	Cor Total	7,329,000	16			
	*R* ^2^	0.9993				
	Adjusted *R* ^2^	0.9984				

^ns^ non‐significant at .05 level

* significant at 5%

** significant at 1%.

**FIGURE 2:**
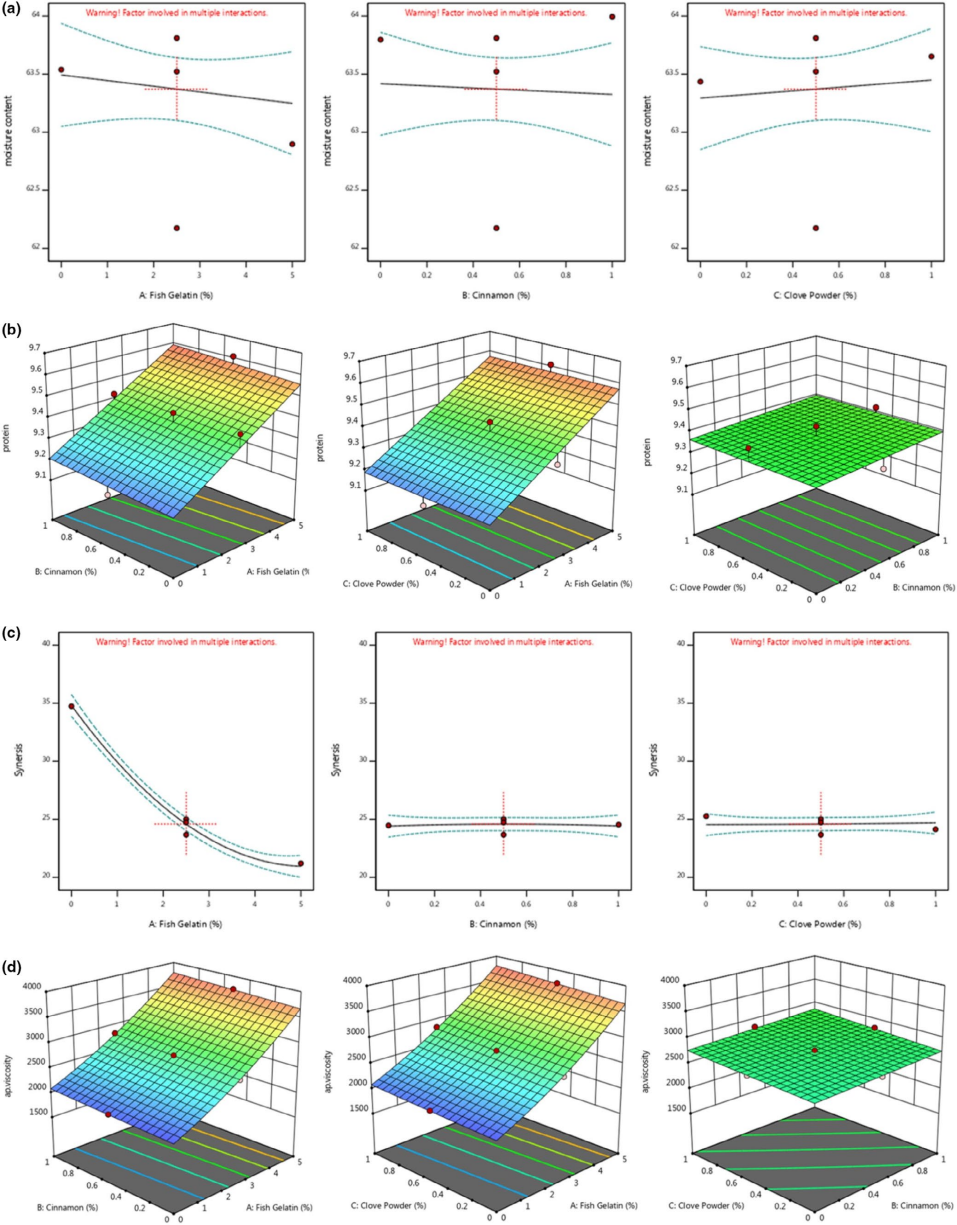
3d surface plots showing the effect of fish gelatin and cinnamon and clove powder on (a) moisture content, (b) protein content, (c) syneresis, and (d) apparent viscosity of pudding formulation

According to Figure 2a, with increasing the fish/bovine gelatin ratio to 3%, the moisture content increased slightly and then decreased significantly by increasing the fish/bovine gelatin ratio to 5%. Increasing the cinnamon powder to 0.5% reduced the moisture content, while the moisture changes in the samples containing clove powder were not significant (*p* > .05). In other words, these changes are constant and linear. However, the moisture content in all treatments was within the standard range (Khanum et al., 2001), so it was possible to keep them at refrigerator temperature. Hartel et al. (2018) suggested that foods with higher moisture content should be stored with relative humidity (RH) of 55%–65% to increase their shelf life (Hartel et al., 2018). The results are in accordance with the results of Yin et al (2021) regarding the effect of adding fish gelatin to yogurt.

### 3.2 Protein content

It is well known that the physical and functional properties of gelatin depend not only on their amino acid composition but also on their Bloom index (Chou et al., 2016), molecular weight distribution, on the relative contents of α‐, β‐, and γ‐chains, and on the presence of protein fragments of low molecular weight (da Trindade Alfaro et al., 2015). It should be noted that modified fish gelatin with anionic polysaccharides from seaweed κ‐carrageenan and gellan gum can be a good alternative to mammalian gelatin (porcine or bovine) in the food industry (Sow et al., 2018). According to Table 3 and the Equation 3, the effect of fish gelatin on the protein contents was quite significant, while other variables had a nonsignificant effect (*p* > .05).

(3) $$\mathrm{Protein}=9.169+0.0776A+0.037B+1.14\times 10^{‐15C}$$

According to Figure 2b, increasing the ratio of fish gelatin to bovine gelatin in the formulation of pudding samples reduced the protein content; in other words, the effect of bovine gelatin on the protein content was significantly greater than fish gelatin. This is due to the low protein content in fish gelatin in comparison with bovine gelatin, which has affected the protein content of the control sample. However, there was a significant difference between protein content of the treatments (*p* < .05). The effects of cinnamon and clove powder addition from 0.5% to 1% on the protein content of samples were ascending and descending, respectively (Figure 2b); this is the case with cinnamon and cloves due to their protein content, which is not comparable to gelatin. The average protein content in cinnamon and clove powder is 4 and 3.3 g/100 g, respectively (Khanum et al., 2001).

### 3.3 Syneresis percent

Syneresis is defined as the extraction of a liquid from a gel that occurs due to slow gel contraction, and it is affected by time. The study results showed that the greater the syneresis value, the easier the gel formed to release water. Therefore, it is not preferred by the consumers (Draget et al., 2001). According to Subaryono et al. (2010), the product stored at low temperature (e.g., pudding) must have high gel strength and low syneresis (Subaryono, 2010).

(4) $$\begin{array}{c} \mathrm{Syneresis}=+34.14‐5.26A+1.162B+0.628C‐0.12\mathrm{AB}\\ \;‐0.17\mathrm{AC}‐0.33\mathrm{BC}+0.53A^{2}‐0.682B^{2}+0.12C^{2} \end{array}$$

It was observed that the experimental data of syneresis for the different formulations and batches did not satisfy equal variances and normality tests (Celeghin et al., 2016). This is a successful outcome because syneresis is a visual sensory attribute that strongly determines the acceptability of this type of products (Mleko & Gustaw, 2002).

According to Table 3 and the Equation 4, the effect of fish gelatin (A) linearly and quadratic (A^2^) on the syneresis changes in pudding samples was quite significant and ascending (*p* < .05), while in the case of other independent variables, this effect was considered nonsignificant (*p* > .05). According to Figure 2c, with increasing the bovine to fish gelatin, syneresis showed a significant decrease (*p* < .05), while the effects of cinnamon and clove powder on syneresis of pudding samples were non‐significant and linear (*p* > .05). The amount of proteins and polysaccharides allowed a positive interaction between them, avoiding undesirable phenomena as the phase separation or coacervation (De Kruif & Tuinier, 2001).

The pudding mixture could be considered a bypass system, where herbal powder includes clove and cinnamon powder located in the network and its concentration increases as starch granules swell by absorbing water after heating. The results were in accordance with the results of Yin et al (2021) regarding the effect of adding fish gelatin to yogurt.

### 3.4 Apparent viscosity

According to Table 3 and the Equation 5, the effect of fish gelatin (A) linearly and quadratic (A^2^) on the viscosity of pudding samples was quite significant and ascending (*p* < .05), while in the case of other independent variables (clove and Cinnamon Powder) only their linear effect was significant (*p* < .05). According to Figure 2d, with increasing the bovine to fish gelatin, viscosity showed a significant increase (*p* < .05), while the cinnamon and clove powder led to a slight increase in viscosity of pudding samples linearly (*p* < .05).

(5) $$\begin{array}{c} \mathrm{Viscosity}=+1947.15+230.904A+79.52B+76.52C+4.0\mathrm{AB}\\ \;+10.0\mathrm{AC}+20.0\mathrm{BC}+20.26A^{2}+6.48B^{2}+6.48C^{2} \end{array}$$

### 3.5 Color parameters

#### 3.5.1 Brightness (L^*^ value)

Color is an important quality characteristic that contributes to the sensorial acceptability of food (García‐Esteban et al., 2003). However, color is affected by many factors such as spices added, packaging, or processing. According to Table 4 and the proposed model (Equation 6), the cinnamon (B) and clove (C) powder had a significant effect on the brightness (L^*^ value) changes linearly (*p* < .05), while the effect of other variables on the L^*^ value was insignificant (*p* > .05). Firdausni et al. (2011) also stated that the cinnamon color intensity comes from tannins (Firdausni & Diza, 2011). In the study of Hassan et al. (2016), the samples of yogurt produced with Moringa powder had a lower L^*^ value, in comparison with samples produced in the research by Shokery et al. (2017) (Hassan et al., 2016; Shokery et al., 2017).

**TABLE 4:** The analysis of variance of the predicted linear and quadratic polynomial models for predicting color parameters of pudding formulation

Response	Source	Sum of squares	*df*	Mean square	*F*‐value	*p*‐Value
L^*^	Model	476.09	3	158.70	765.50	<.0001*
	A‐Fish/Bovine Gelatin	0.0176	1	0.0176	0.0851	.7751
	B‐Cinnamon Powder	450.38	1	450.38	2172.46	<.0001
	C‐Clove Powder	25.70	1	25.70	123.95	<.0001
	Residual	2.70	13	0.2073		
	Lack of Fit	2.63	11	0.2387	6.84	.1344^ns^
	Pure Error	0.0698	2	0.0349		
	Cor Total	478.78	16			
	*R* ^2^	0.9944				
	Adjusted *R* ^2^	0.9931				
a^*^	Model	528.07	9	58.67	1883.13	<.0001*
	A‐Fish/Bovine Gelatin	0.0102	1	0.0102	0.3286	.5844
	B‐Cinnamon Powder	521.86	1	521.86	16,748.94	<.0001
	C‐Clove Powder	3.79	1	3.79	121.78	<.0001
	AB	0.0496	1	0.0496	1.59	.2474
	AC	0.1891	1	0.1891	6.07	.0432
	BC	0.2278	1	0.2278	7.31	.0305
	A²	0.0916	1	0.0916	2.94	.1301
	B²	1.37	1	1.37	43.97	.0003
	C²	0.0242	1	0.0242	0.7772	.4072
	Residual	0.2181	7	0.0312		
	Lack of Fit	0.2055	5	0.0411	6.52	.1382^ns^
	Pure Error	0.0126	2	0.0063		
	Cor Total	528.29	16			
	*R* ^2^	0.9996				
	Adjusted *R* ^2^	0.9991				
b^*^	Model	2136.79	9	237.42	7103.06	<.0001*
	A‐Fish/Bovine Gelatin	0.0109	1	0.0109	0.3258	.5860
	B‐Cinnamon Powder	2090.34	1	2090.34	62,537.88	<.0001
	C‐Clove Powder	2.98	1	2.98	89.19	<.0001
	AB	0.0190	1	0.0190	0.5688	.4753
	AC	0.0091	1	0.0091	0.2726	.6177
	BC	0.1035	1	0.1035	3.10	.1218
	A²	0.0047	1	0.0047	0.1407	.7187
	B²	27.21	1	27.21	814.10	<.0001
	C²	0.0120	1	0.0120	0.3588	.5681
	Residual	0.2340	7	0.0334		
	Lack of Fit	0.1762	5	0.0352	1.22	.5080^ns^
	Pure Error	0.0578	2			
	Cor Total	2137.02	16			
	*R* ^2^	0.9999				
	Adjusted *R* ^2^	0.9997				

^ns^ non‐significant at .05 level

* Significant at .05 level.

**FIGURE 3:**
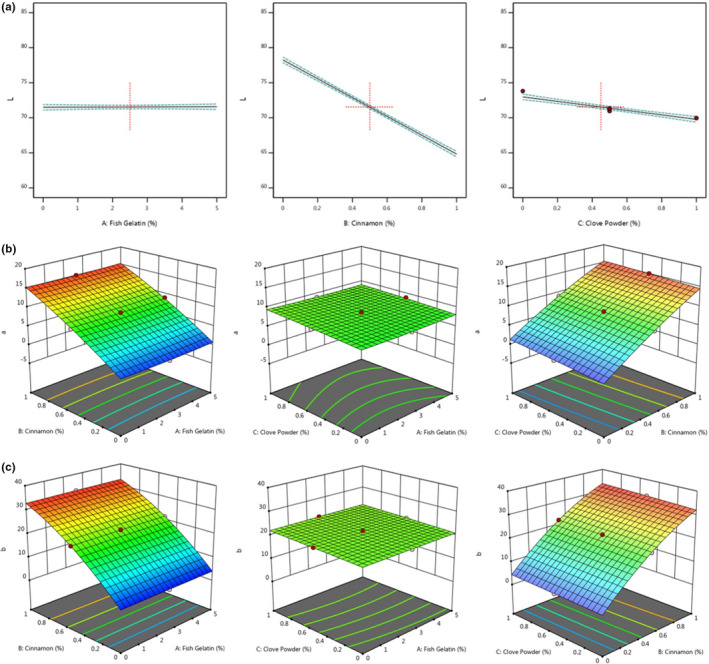
3d surface plots showing the effect of fish gelatin and cinnamon and clove powder on color parameters: (a) L*, (b) a*, and (c) b* of pudding formulation

According to Figure 3a, with increasing the cinnamon and clove powder, the light intensity of the pudding samples was decreasing, but in cinnamon powder, this reduction had a more slope (Figure 3a). The decreasing L^*^ value shows that the specimen underwent a color change to a darker color. It is also possible to change the arrangement of milk caseins with retentate, which can create a denser structure and more chain linkages by accumulation, and increase the L^*^ value (Aghajani et al., 2019).

(6) L=+79.65553+0.016800A‐13.42200B‐3.20600C

#### 3.5.2 Redness (a^*^ value)

Authors such as Dvorak et al., (2001) concluded that a* value was the most important aspect of color (Dvorak et al., 2001). According to Table 4 and Figure 3b, cinnamon and clove powder linearly, interactions of gelatins–clove powder and cinnamon–clove powder, and the quadratic model of cinnamon powder had a significant effect on redness (a^*^) changes (*p* < .05) and its predictive equation can be found as Equation 7.

(7) $$\begin{array}{c} a=‐0.0798‐0.131A+17.488B+2.257C+0.063\mathrm{AB}‐0.123\mathrm{AC}\\ \;‐0.675\mathrm{BC}+0.0296A^{2}‐2.86B^{2}‐0.38C^{2} \end{array}$$

Studies of Shihabudeen et al. (2011) also confirm the presence of flavonoids, tannins, saponins, steroid, glycosides, coumarins, anthraquinones, and alkaloids in the cinnamon bark extract (Shihabudeen et al., 2011). The greatest change in the a^*^ value serves to turn the specimen a more reddish color than the initial value. The red color comes from the reddish‐brown color of the cinnamon (Firdausni & Diza, 2011).

#### 3.5.3 Yellowness (b^*^ value)

According to Table 4, Figure 3c, and the Equation 8, the linear effects of cinnamon (B) and clove (C) powder and cinnamon powder square (B^2^) on yellowness were significant (*p* < .05). Some constituents frequently encountered in cinnamon include procyanidins and phenolic acids. Both cinnamon and clove contain phenolic compounds and flavonoids and tannins that produce a yellow color, have a water‐soluble characteristic, does not crystallize, and mix with proteins from the suspension (Kusstianti et al., 2017).

(8) $$Y=21.66+14.45B+0.545C‐3.18B^{2}$$

The greatest observed change in the b^*^ value was related to the spices containing samples that may be due to the physical color properties of tannin, which range from clear yellowish to light brown (Anggono et al., 2018). In addition, cinnamon also contains 55%–65% cinnamaldehyde, which has a physical yellowish color. Thus, the content of cinnamaldehyde likely also affects the color of tooth enamel (Wijayanti et al., 2009).

#### 3.5.4 Sensory evaluation

**FIGURE 4:**
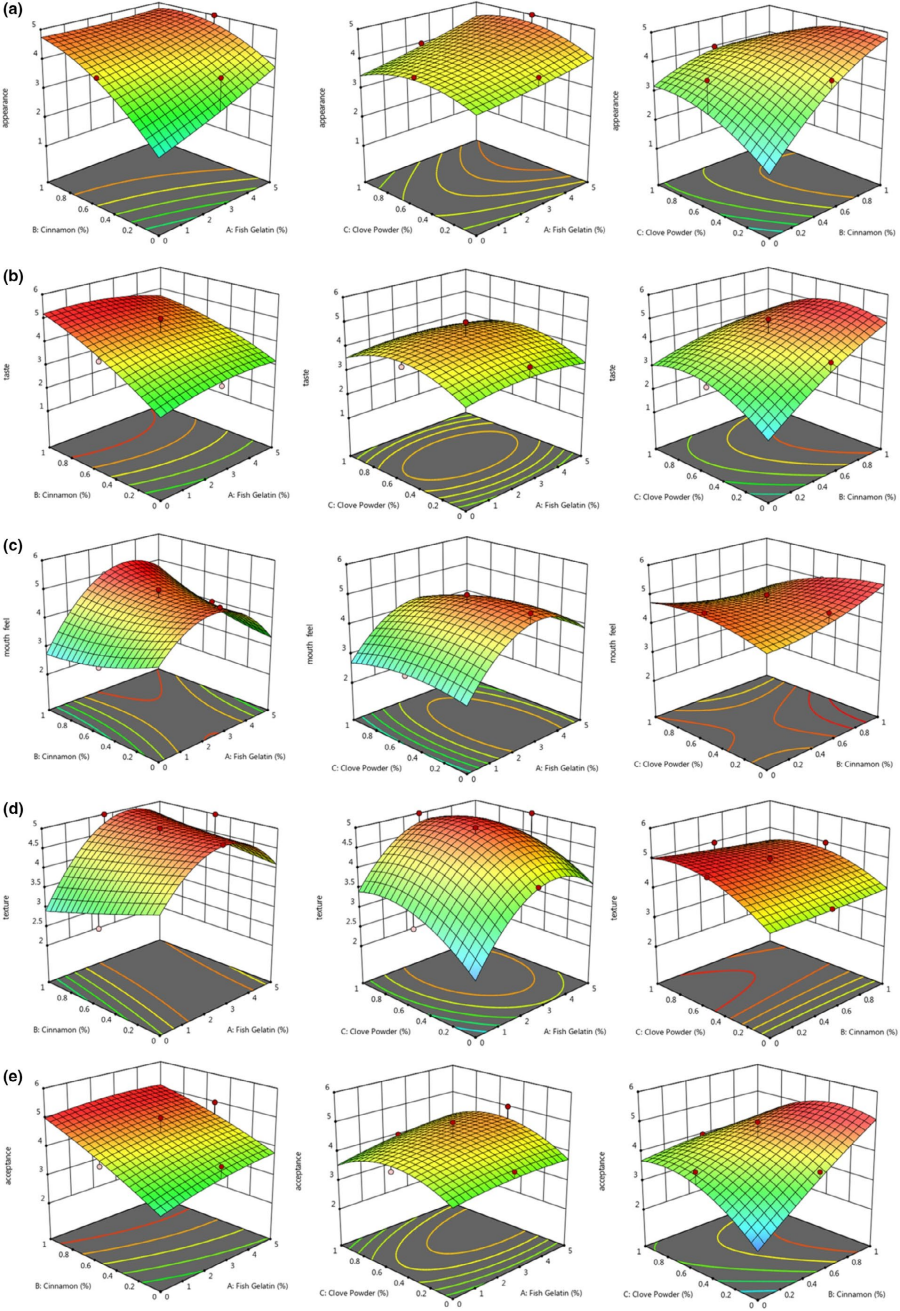
3d surface plots showing the effect of fish gelatin and cinnamon and clove powder on sensory attributes: (a) appearance, (b) taste, (c) mouthfeel, (d) texture, and (e) overall acceptance of pudding formulation

Figure 4 and Table 5 showed the analysis of variance and variation of sensory attributes of producing pudding formulation under the effects of fish/bovine gelatin (A), cinnamon powder (B), and clove (C) powder, respectively. The variation of sensory attributes of pudding formulation are as following.

**TABLE 5:** The analysis of variance of the predicted linear and quadratic polynomial models for predicting sensory attributes of pudding formulation

Response	Source	Sum of squares	*df*	Mean square	*F*‐value	*p*‐Value
Appearance	Model	16.43	9	1.83	4.86	0.0245^*^
	A‐Fish Gelatin	1.60	1	1.60	4.26	0.0778
	B‐Cinnamon Powder	8.10	1	8.10	21.58	0.0024
	C‐Clove Powder	0.1000	1	0.1000	0.2664	0.6217
	AB	1.12	1	1.12	3.00	0.1270
	AC	0.1250	1	0.1250	0.3330	0.5820
	BC	3.13	1	3.13	8.32	0.0235
	A²	0.0085	1	0.0085	0.0227	0.8846
	B²	0.5274	1	0.5274	1.40	0.2746
	C²	0.5274	1	0.5274	1.40	0.2746
	Residual	2.63	7	0.3754		
	Lack of Fit	2.63	5	0.5256		
	Pure Error	0.0000	2	0.0000		
	Cor Total	19.06	16			
	*R* ^2^	0.8621				
	Adjusted *R* ^2^	0.6848				
Taste	Model	17.07	9	1.90	5.39	0.0185^*^
	A‐Fish Gelatin	0.0000	1	0.0000	0.0000	1.0000
	B‐Cinnamon Powder	10.00	1	10.00	28.42	0.0011
	C‐Clove Powder	0.1000	1	0.1000	0.2842	0.6105
	AB	0.5000	1	0.5000	1.42	0.2721
	AC	0.0000	1	0.0000	0.0000	1.0000
	BC	2.00	1	2.00	5.68	0.0486
	A²	0.1536	1	0.1536	0.4365	0.5300
	B²	0.1536	1	0.1536	0.4365	0.5300
	C²	1.46	1	1.46	4.16	0.0807
	Residual	2.46	7	0.3519		
	Lack of Fit	1.80	5	0.3593	1.08	0.5457^ns^
	Pure Error	0.6667	2	0.3333		
	Cor Total	19.53	16			
	*R* ^2^	0.8739				
	Adjusted *R* ^2^	0.7117				
Mouthfeel	Model	13.85	9	1.54	9.83	0.0032^**^
	A‐Fish Gelatin	1.60	1	1.60	10.22	0.0151
	B‐Cinnamon Powder	0.1000	1	0.1000	0.6390	0.4503
	C‐Clove Powder	0.4000	1	0.4000	2.56	0.1539
	AB	3.13	1	3.13	19.97	0.0029
	AC	0.1250	1	0.1250	0.7988	0.4011
	BC	1.13	1	1.13	7.19	0.0315
	A²	4.40	1	4.40	28.13	0.0011
	B²	0.1277	1	0.1277	0.8160	0.3964
	C²	0.2126	1	0.2126	1.36	0.2820
	Residual	1.10	7	0.1565		
	Lack of Fit	0.4288	5	0.0858	0.2573	0.9042^ns^
	Pure Error	0.6667	2	0.3333		
	Cor Total	14.94	16			
	*R* ^2^	0.9267				
	Adjusted *R* ^2^	0.8324				
Texture	Model	12.32	9	1.37	5.71	0.0158^**^
	A‐Fish Gelatin	2.50	1	2.50	10.43	0.0145
	B‐Cinnamon Powder	0.1000	1	0.1000	0.4170	0.5390
	C‐Clove Powder	1.60	1	1.60	6.67	0.0363
	AB	1.12	1	1.12	4.69	0.0670
	AC	0.1250	1	0.1250	0.5213	0.4937
	BC	0.1250	1	0.1250	0.5213	0.4937
	A²	2.57	1	2.57	10.71	0.0136
	B²	0.0012	1	0.0012	0.0050	0.9457
	C²	0.6144	1	0.6144	2.56	0.1535
	Residual	1.68	7	0.2398		
	Lack of Fit	1.01	5	0.2024	0.6071	0.7179^ns^
	Pure error	0.6667	2	0.3333		
	Cor total	14.00	16			
	*R* ^2^	0.8801				
	Adjusted *R* ^2^	0.7260				
Overall acceptance	Model	12.56	9	1.40	6.80	0.0096^**^
	A‐Fish Gelatin	0.4000	1	0.4000	1.95	0.2053
	B‐Cinnamon Powder	6.40	1	6.40	31.19	0.0008
	C‐Clove Powder	0.1000	1	0.1000	0.4874	0.5076
	AB	0.1250	1	0.1250	0.6092	0.4607
	AC	0.1250	1	0.1250	0.6092	0.4607
	BC	3.12	1	3.12	15.23	0.0059
	A²	0.0340	1	0.0340	0.1658	0.6961
	B²	0.0340	1	0.0340	0.1658	0.6961
	C²	1.01	1	1.01	4.90	0.0624
	Residual	1.44	7	0.2052		
	Lack of Fit	0.7696	5	0.1539	0.4618	0.7898^ns^
	Pure Error	0.6667	2	0.3333		
	Cor Total	14.00	16			
	*R* ^2^	0.8974				
	Adjusted *R* ^2^	0.7655				

^ns^ non‐significant at .05 level

^*^ significant at 5%

^**^ significant at 1%.

#### 3.5.5 Appearance

Sensory evaluation started developing with the growth of industry and processed food (Ruiz‐Capillas et al., 2021). Sensory characteristics are crucial in the development of new food products (Worch et al., 2010) and influence consumer acceptance both before purchase (visual appearance) and at the time of consumption such as odor and flavor. Because of this, sensory analysis is one of the most important methods in judging food quality (Djekic et al., 2021). According to Table 5 and Equation 9, cinnamon powder had a significant effect on the appearance scores (*p* < .05).

(9) $$\begin{array}{c} \mathrm{Appearance}=+1.15+0.22A+5.58B+2.98C‐0.3\mathrm{AB}+0.1\mathrm{AC}\\ \;‐2.5\mathrm{BC}+0.01A^{2}‐1.8B^{2}‐1.8C^{2} \end{array}$$

In other words, cinnamon powder, more than fish gelatin and clove powder, was effective in changing the appearance of pudding samples. Figure 4a also shows that increasing the cinnamon powder to the pudding formulation increased the appearance scores, while in the case of fish gelatin, this was a downward trend. Increasing the level of clove powder led to a nonsignificant increase in appearance scores (Figure 4a). The addition of spices provides new tastes, colors, and aromas for food that even gives culinary identity (De La Torre Torres et al., 2017) owing to the changes in the composition of volatile compounds (Jung et al., 2014) that affect the hedonic characteristics and may affect the acceptance of new products. On the other hand, spices could improve the quality of food products due to their preservative properties (Dini, 2018; Gottardi et al., 2016).

#### 3.5.6 Taste and mouthfeel

Taste and flavor are one of the important parameters in the accepting pudding, which many factors effect on the taste. For example, milk flavor is apparent in products containing higher amounts of fat, such as cream, particularly when it is used warm. According to Equation 10, the linear effect of cinnamon powder (B) and the interaction effect of cinnamon–clove powder (BC) on taste score were significant (*p* < .05), while other variables had no significant effect on the taste of the samples (Figure 4c).

(10) $$\begin{array}{c} \mathrm{Taste}=+1.35+0.292A+4.46B+4.16C‐0.20\mathrm{AB}+2.84\times 10\\ \;‐16\mathrm{AC}‐2.0\mathrm{BC}‐0.04A^{2}‐0.96B^{2}‐2.96C^{2} \end{array}$$

Equation 11 showed that the linear effect of fish gelatin (A), the interaction effect of fish gelatin–cinnamon powder (AB), cinnamon–clove powder (BC), and the quadratic effect of fish gelatin (A^2^) on mouthfeel score were significant (*p* < .05), while other variables had no significant effect on the mouthfeel of the samples (Figure 4c).

(11) $$\begin{array}{c} \mathrm{Mouthfeel}=+3.21+0.99A‐1.17B+1.73C+0.50\mathrm{AB}\\ \;‐0.10\mathrm{AC}‐1.5\mathrm{BC}‐0.21A^{2}+0.87B^{2}‐1.13C^{2} \end{array}$$

The effects of gelatin on the perception of flavor have been investigated in terms of the taste–aroma interaction (Cook et al., 2003). According to Figure 4b, the effects of fish gelatin to bovine gelatin ratio and clove powder on taste scores were nonsignificant (*p* > .05) and with increasing the content of each variable from 2.5% and 0.5%, respectively, the taste score decreased. As can be seen from Figure 4b, by increasing the cinnamon powder from 0% to 1%, the taste score increased significantly (*p* < .05). As can be seen from Figure 4C, by increasing fish/bovine gelatin ratio from 0% to 3% the mouthfeel score increased, but decreased at higher than 3% fish/bovine gelatin ratio. As can be seen, cinnamon powder incensement leads to a slight increase in the mouthfeel score, while clove powder addition decreased mouthfeel score slightly (*p* > .05).

#### 3.5.7 Texture

Texture is an important attribute in dairy desserts such as pudding, since it is highly correlated with consumer acceptance. Gelatin with a higher gel strength will produce a harder jelly. The main factors affecting functional properties of starch gel are as follows: amylose content and the rigidity of the amylose matrix, flexibility of the remaining swollen/ungelatinized starch granules, and their interactions. Thus, gel hardness must be related to amylose matrix as well as to the filling effect of swollen starch granules (Bierzuńska et al., 2019). However, De Wijk et al. (2003) and Weenen et al. (2003) observed that in custard desserts texture is a multimodal attribute that can be affected not only by textural attributes (thick, smooth, fatty, rough, and grainy) but also by some taste/flavor attributes. According to Equation 12, the linear effect of fish gelatin (A) and clove powder (C) as well as the squared cinnamon (A^2^) on the pudding texture was effective (*p* < .05). Due to the protein nature of fish and cow gelatin, changes in the texture of pudding samples can be affected by gelatin changes (De Wijk et al., 2003; Weenen et al., 2003).

(12) $$\begin{array}{c} \mathrm{Texture}=+2.73+0.883A‐0.785B+3.22C+0.30\mathrm{AB}\\ \;‐0.10\mathrm{AC}‐0.50\mathrm{BC}‐0.16A^{2}+0.085B^{2}‐1.92C^{2} \end{array}$$

As can be seen from Figure 4d, by increasing the ratio of fish gelatin to bovine gelatin from 0% to 2.5%, the texture scores increased, but after that, the scores decreased significantly. In the case of cinnamon powder, this trend was linear and concentration of 0.5% was introduced as the optimal point. Increasing the clove powder from 0% to 0.6%, increased the texture scores and then decreased. De Wijk et al. (2003) and Weenen et al. (2003) observed that in custard desserts texture is a multimodal attribute that can be affected not only by textural attributes (thick, smooth, fatty, rough, grainy) but also by some taste/flavor attributes such as the mucilage, polysaccharides, which are available in cinnamon.

#### 3.5.8 Overall acceptance

According to Equation 13, the linear effect of cinnamon powder (B) and the interaction effect of cinnamon–clove powder (BC) on the overall acceptance were significant (*p* < .05). Therefore, compared to gelatin, the overall acceptance was more affected by spices powder (Table 5). According to Figure 4E, with increasing the cinnamon powder, the overall acceptance also increased significantly (*p* < .05), while in the case of clove powder, the maximum effect on the overall acceptance was 0.5%. As the ratio of fish gelatin to bovine gelatin increased, the overall acceptance decreased significantly. Panelists preferred samples containing cinnamon and clove powder than gelatins (Figure 4e).

(13) $$\begin{array}{c} \mathrm{Overall}\mathrm{acceptance}=+1.93+0.17A‐3.55B+3.65C‐0.10\mathrm{AB}+0.10\mathrm{AC}\\ \;‐2.50\mathrm{BC}‐0.018A^{2}‐0.45B^{2}‐2.45C^{2} \end{array}$$

In the study of Abdo Qasem et al. (2017), seedless okra pods were added to pudding at 0%, 2%, 4%, 6%, and 8%. The overall acceptance showed that 2% okra pods were closer to the control in terms of overall acceptability (Abdo Qasem et al., 2017).

#### 3.5.9 Optimization

**FIGURE 5:**
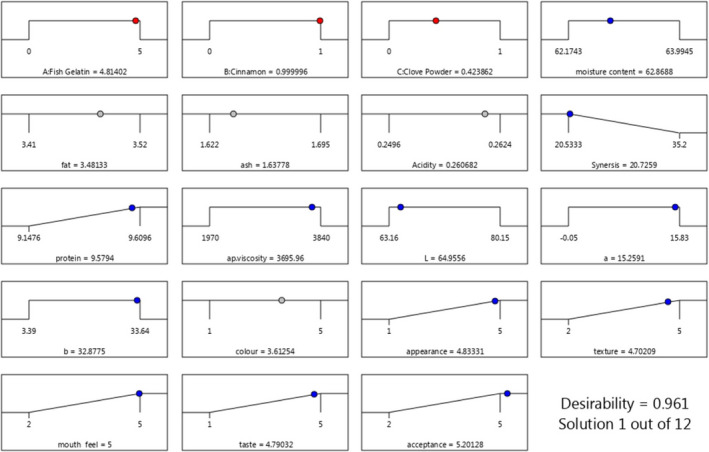
The optimum conditions for producing pudding formulation containing fish gelatin and clove and cinnamon powder

To select the optimization conditions, the amount of fat and syneresis should be minimal while the amount of protein and sensory parameters should be maximum. Other responses should be in the range. Figures 5 show the optimal levels of additives including the ratio of fish gelatin to bovine gelatin, cinnamon and clove powder, and the obtained optimum response parameters from the response surface methodology with desirability of 0.961.

## 4 CONCLUSIONS

These results indicate that the acceptability of the formulated samples may be improved simply by changing the concentrations of flavoring agents and fish gelatin. This study demonstrates that it is feasible to replace bovine gelatin by fish gelatin in puddings formulation, although it is necessary to improve the sensory profile to reach a better acceptability. In general, it should be noted that fish gelatin can be a good alternative to mammalian gelatin (porcine or bovine) in the food industry. Also, the use of spices such as cinnamon and clove powder can play an important role in improving quality characteristics, especially sensory scores.

## 5 COMPLIANCE WITH ETHICS REQUIREMENTS

This article does not contain any studies with human or animal subjects.

## CONFLICT OF INTEREST

None.

## Data Availability

The authors confirm that the data supporting the findings of this study are available within the article.
